# Mitochondrial-Targeted Antioxidant Maintains Blood Flow, Mitochondrial Function, and Redox Balance in Old Mice Following Prolonged Limb Ischemia

**DOI:** 10.3390/ijms18091897

**Published:** 2017-09-04

**Authors:** Shunsuke Miura, Shu-ichi Saitoh, Tomoki Kokubun, Takashi Owada, Hiroyuki Yamauchi, Hirofumi Machii, Yasuchika Takeishi

**Affiliations:** Cardiovascular Medicine, Fukushima Medical University, 1 Hikarigaoka, Fukushima 960-1295, Japan; eggcello@fmu.ac.jp (S.M.); kokutomo@fmu.ac.jp (T.K.); to927@fmu.ac.jp (T.O.); hymuc@fmu.ac.jp (H.Y.); machii@fmu.ac.jp (H.M.); takeishi@fmu.ac.jp (Y.T.)

**Keywords:** oxidative stress, angiogenesis, mitochondria, ischemia, p53, proliferator-activated receptor γ coactivator-1α (PGC-1α), aging

## Abstract

Aging is a major factor in the decline of limb blood flow with ischemia. However, the underlying mechanism remains unclear. We investigated the role of mitochondrial reactive oxygen species (ROS) with regard to limb perfusion recovery in aging during ischemia. We performed femoral artery ligation in young and old mice with or without treatment with a scavenger of mitochondrial superoxide, MitoTEMPO (180 μg/kg/day, from pre-operative day 7 to post-operative day (POD) 21) infusion using an implanted mini-pump. The recoveries of cutaneous blood flow in the ischemic hind limb were lower in old mice than in young mice but were improved in MitoTEMPO-treated old mice. Mitochondrial DNA damage appeared in ischemic aged muscles but was eliminated by MitoTEMPO treatment. For POD 2, MitoTEMPO treatment suppressed the expression of p53 and the ratio of Bax/Bcl2 and upregulated the expression of hypoxia-inducible factor-1α (HIF-1α) and vascular endothelial growth factor (VEGF) in ischemic aged skeletal muscles. For POD 21, MitoTEMPO treatment preserved the expression of PGC-1α in ischemic aged skeletal muscle. The ischemic soleus of old mice showed a lower mitochondrial respiratory control ratio in POD 21 compared to young mice, which was recovered in MitoTEMPO-treated old mice. Scavenging of mitochondrial superoxide attenuated mitochondrial DNA damage and preserved the mitochondrial respiration, in addition to suppression of the expression of p53 and preservation of the expression of peroxisome proliferator-activated receptor γ coactivator-1α (PGC-1α) in ischemic skeletal muscles with aging. Resolution of excessive mitochondrial superoxide could be an effective therapy to recover blood flow of skeletal muscle during ischemia in senescence.

## 1. Introduction

Senescence is an irreversible risk factor for most forms of cardiovascular and peripheral artery disease (PAD) [1,2,3]. Previous reports demonstrated aging-dependent impairment of blood flow with critical limb ischemia [4,5]. However, this mechanism remains complicated. Oxidative stress increase with age is one of the causes of vascular dysfunction [6]. Age-dependent increases in oxidative stress occur through multiple mechanisms, including mitochondrial dysfunction. The mitochondria are major sources of reactive oxygen species, which are increased in aging animals [7,8]. This increase in mitochondrial reactive oxygen species (ROS) is associated with the oxidation of the electron transport system (ETS) complex V, leading to decreased ATP production [9,10] and increased DNA oxidative damage [9,11]. Moreover, in PAD patients, mitochondrial dysfunction and increased oxidative stress-induced damage have been reported [12,13]. Taken together, it seems that the resolution of mitochondrial ROS in aged mammals is attributed to collateral growth under ischemia through the recovery of mitochondria or other signaling pathways. In contrast, transient or low levels of ROS also act as mediators of angiogenesis through the hypoxia-inducible factor (HIF)-vascular endothelial growth factor (VEGF)/VEGF receptor-2 signaling pathway and the VEGF-independent pathway, the latter of which involves the generation of lipid oxidation products [14]. Therefore, we performed the present study to examine whether mitochondrial ROS scavenging maintains blood flow following acute hind limb ischemia in aged mice. In this study, limb perfusion recovery in old mice treated with MitoTEMPO was observed compared to young and old mice. MitoTEMPO is a superoxide scavenger accumulated in the mitochondria [15]. MitoTEMPO was previously used in vivo in mice to treat hypertension-induced endothelial dysfunction [15,16]. We demonstrated the mechanism of angiogenesis with regard to p53 and the peroxisome proliferator-activated receptor γ coactivator-1α (PGC-1α) pathway as well as HIF-VEGF signaling. In addition, we elucidated the effect of mitochondrial ROS in aging with respect to mitochondrial DNA (mtDNA) and apoptosis.

## 2. Results

### 2.1. The Levels of Mitochondrial H_2_O_2_ and Superoxide in Skeletal Muscle

**Figure 1:**
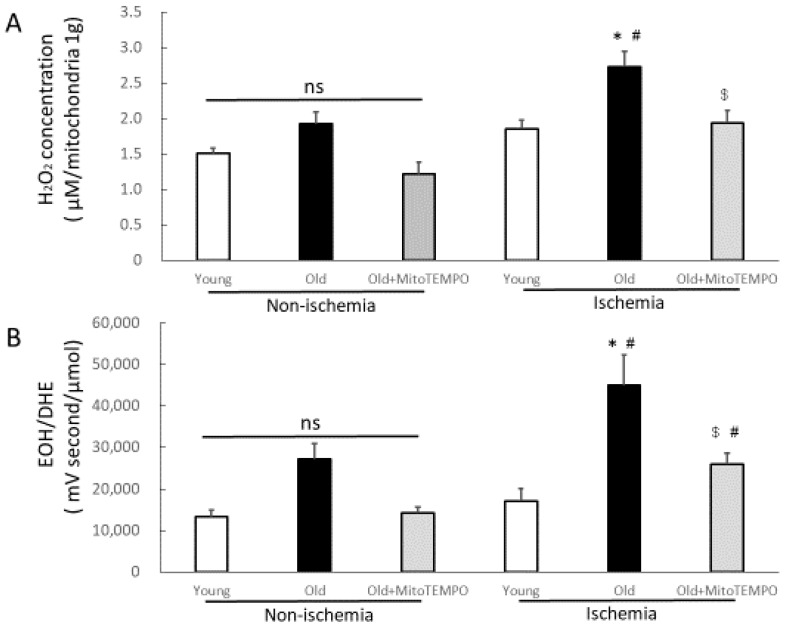
Effect of MitoTEMPO on mitochondrial H_2_O_2_ and superoxide in tissue extracts of ischemic skeletal muscle. The mitochondrial H_2_O_2_ concentration was measured in non-ischemic and ischemic (post-operative day (POD) 2) skeletal muscle from young, old, and MitoTEMPO-treated old mice (**A**); the levels of superoxide in tissue extracts were determined by the ultra-performance liquid chromatography (UPLC) method (**B**). The levels of mitochondrial H_2_O_2_ and superoxide in tissue extracts under ischemic conditions were higher in old mice than in young mice from the ischemic group. MitoTEMPO treatment decreased the levels of mitochondrial H_2_O_2_ and superoxide in tissues to levels of young mice. The values are expressed as the means ± S.E.M. *n* = 10, each. * *p* < 0.05 vs. young mice, $ *p* < 0.05 vs. old mice, # *p* < 0.05 vs. non-ischemic skeletal muscle. ns indicates no significant difference.

Because the increase in mitochondrial ROS is assumed to be a major factor in the decline of blood flow with ischemia, we initially measured the level of mitochondrial H_2_O_2_ in the non-ischemic and ischemic skeletal muscles of young, old, and MitoTEMPO-treated old mice in post-operative day (POD) 2. There was no significant difference among the three groups in non-ischemic skeletal muscles. Under ischemic conditions, the level of mitochondrial H_2_O_2_ was elevated in only the old mice compared to non-ischemic conditions. Moreover, the level of mitochondrial H_2_O_2_ was higher in the old mice than in young mice under ischemic conditions. MitoTEMPO treatment decreased the level of mitochondrial H_2_O_2_ to the level of young mice (Figure 1A). Since mitochondria are the major source of ROS in aging tissues [17,18]. Mitochondrial-targeted antioxidants may contribute to decreased ROS in tissues. To determine whether MitoTEMPO could reduce ROS in tissues, we measured the levels of superoxide in the hind limb by the ultra-performance liquid chromatography (UPLC) method. The levels of superoxide in non-ischemic hind limb tissue did not differ among the three groups. The level of superoxide was elevated in the ischemic hind limb of old mice compared to non-ischemic mice (*p* = 0.029). MitoTEMPO-treated old ischemic muscle also had a tendency to have a higher superoxide level than non-ischemic muscle, though these changes were not statistically significant (*p* = 0.293). The levels of superoxide in tissue extracts under ischemia were higher in old mice than in young mice, and those were attenuated by MitoTEMPO treatment (44,948.1 ± 7266.3 vs. 17,008.5 ± 3133.1 and 26,004.0 ± 2234.3 mVs/μmol, respectively, *n* = 10, *p* < 0.05; Figure 1B).

### 2.2. Limb Blood Flow Recovery

**Figure 2:**
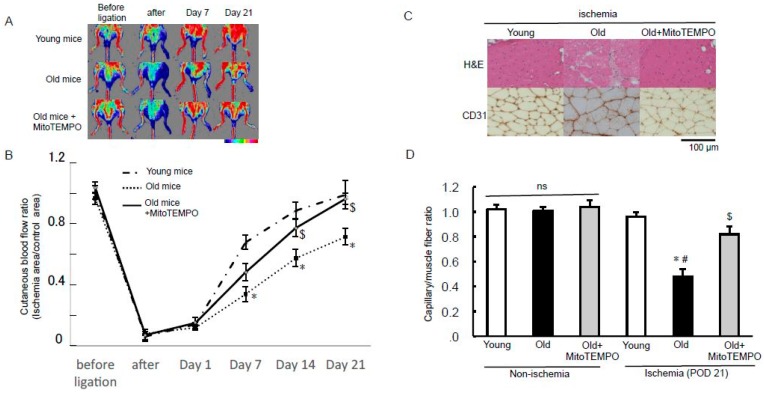
Effect of MitoTEMPO on age-related impairment of angiogenesis after ischemia. Representative hind limb perfusion imaging after inducing hind limb ischemia in young, old, and MitoTEMPO-treated old mice (**A**); ischemic/non-ischemic limb blood flow ratio was used for quantitative analysis (*n* = 10, each. * *p* < 0.05 vs. young mice, $ *p* < 0.05 vs. old mice) (**B**); representative sections after H & E staining and immunohistochemical staining. Immunohistochemical staining was performed using anti-CD31 antibody to determine the capillary density of ischemic muscle on POD 21 (**C**); quantification of capillary density was shown as the capillary/muscle fiber ratio (*n* = 10, each. * *p* < 0.05 vs. young mice, $ *p* < 0.05 vs. old mice, # *p* < 0.001 vs. old mice in non-ischemia) (**D**). The values are expressed as the means ± S.E.M. ns indicates no significant difference.

We examined the effect of MitoTEMPO on age-related impairment of limb perfusion recovery using laser Doppler perfusion imaging. The blood flow recovery was more impaired in the ischemic hind limb of old mice than in those of young mice. MitoTEMPO treatment improved the blood flow recovery (Figure 2A,B). The improvement of blood flow recovery was reflected by an increase in capillary density as determined by CD 31 staining of the gastrocnemius and soleus muscles (Figure 2C,D). These data suggest that mitochondrial ROS scavenging recovers the age-related impairment of angiogenesis.

### 2.3. mtDNA Damage

**Figure 3:**
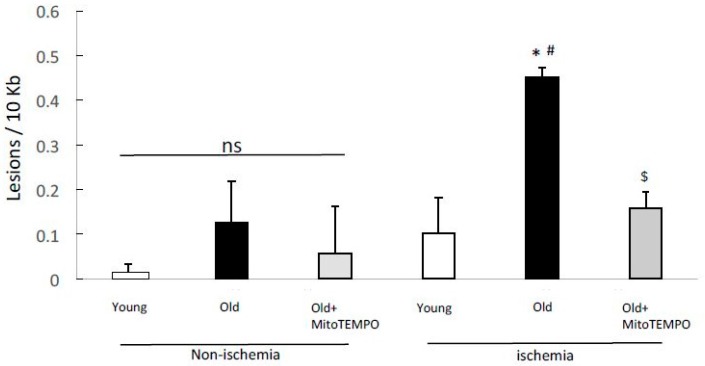
Effect of MitoTEMPO on mtDNA damage after ischemia induction. mtDNA damage was increased in old mice compared to young and MitoTEMPO-treated old mice 2 days after ischemia induction, but not in non-ischemic muscles. The values are expressed as the means ± S.E.M. *n* = 10, each. * *p* < 0.05 vs. young mice, $ *p* < 0.05 vs. old mice, # *p* < 0.05 vs. non-ischemic muscle. ns indicates no significant difference.

mtDNA is highly susceptible to oxidative stress because of the absence of protective histones, leading to more vulnerable nuclear DNA. Mitochondrial ROS increase with age and are associated with oxidative mitochondrial DNA damage and endothelial/vascular dysfunction [19]. To examine whether MitoTEMPO treatment prevented the mtDNA damage induced by aging and ischemia, we performed different length quantitative PCR for the mtDNA of both ischemic and non-ischemic skeletal muscles. mtDNA damage was increased in old mice compared to young mice and MitoTEMPO-treated old mice (0.45 ± 0.02 vs. 0.10 ± 0.08 and 0.15 ± 0.03 lesions/10 kb, *n* = 10, *p* < 0.05, respectively) after ischemia but not in non-ischemic skeletal muscles on POD 2 (Figure 3). These results suggest that mtDNA damage induced by ischemia increases with senescence, but is suppressed by mitochondrial superoxide scavenging.

### 2.4. Respiratory Profile of Mitochondria in Skeletal Muscle

**Figure 4:**
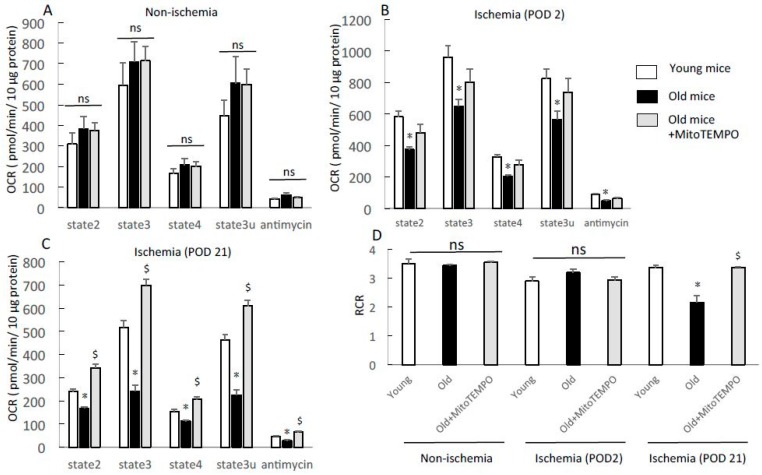
Respiration profile from isolated mitochondria in soleus. The mitochondrial respiration profile was assessed using the XF24 flux analyzer in non-ischemic and ischemic (POD 2 and 21) skeletal muscles of young, old, and MitoTEMPO-treated old mice. States 2, 3, 4, and 3u respiration and response to antimycin in non-ischemic skeletal muscles did not differ among the three groups of mice (**A**); states 2, 3, 4, and 3u respiration and response to antimycin in ischemic skeletal muscles (POD 2) were lower in old mice than that in young mice. MitoTEMPO treatment preserved mitochondrial respiration, though it was not significantly different (**B**); states 2, 3, 4, and 3u respiration and response to antimycin in ischemic soleus (POD 21) were lower in old mice than that of young mice. MitoTEMPO treatment preserved mitochondrial respiration (**C**); the respiratory control ratio (RCR: state 3/state 4 respiration in the presence of succinate) of ischemic soleus (POD 21) was lower in old mice than in young and MitoTEMPO-treated old mice, whereas that of non-ischemic and ischemic (POD 2) skeletal muscles did not differ among the three groups (**D**). The values are expressed as the means ± S.E.M. *n* = 10, each. * *p* < 0.05 vs. young mice, $ *p* < 0.05 vs. old mice. ns indicates no significant difference.

It has been controversial that whether aging is related to impairment of mitochondrial respiration in skeletal muscle [20,21,22,23,24]. How ischemia alters the mitochondrial respiration profile in aging skeletal muscle is not fully understood. Moreover, the effect of mitochondrial ROS scavenging for ischemic skeletal muscle is unclear. Thus, we examined the change in the mitochondrial respiration profile of ischemic skeletal muscles using a XF24 flux analyzer (Seahorse Bioscience, North Billerica, MA, USA). We chose the soleus to minimize errors because mitochondrial respiratory function is muscle type specific and the soleus is primarily composed of slow twitch fibers [25]. In non-ischemic skeletal muscles, mitochondrial respiration via complex II, including states 2, 3, 4, and 3u and in response to antimycin A, did not differ among young, old, and Mito-TEMPO-treated old mice (Figure 4A). In ischemic skeletal muscles of POD 2, the respiration rate of each state was higher in young mice than old mice (Figure 4B). MitoTEMPO-treated old mice had a tendency to have higher respiration rates compared to non-treated one. In ischemic skeletal muscles at POD 21, the respiration rate of each state was higher in young mice than in old mice. Furthermore, MitoTEMPO treatment significantly preserved mitochondrial respiration compared to no treatment (Figure 4C). These data suggest that MitoTEMPO treatment in old mice could preserve mitochondrial respiration under ischemia to a similar level as that of young mice. The respiratory control ratio (RCR, state 3/state 4) is an index of coupling for diagnosis of ETS defects, indicating the efficiency of ATP production in the presence of ADP. In ischemic skeletal muscles at POD 21, the mitochondria of old mice displayed a lower RCR value than those of young mice, and MitoTEMPO treatment elevated RCR value to the level as young mice (Figure 4D). The RCR value of both non-ischemic and ischemic skeletal muscles at POD 2 revealed no differences among the three groups (Figure 4D). These data suggested that MitoTEMPO treatment for old mice might have contributed to the preservation of ATP production through ETS in chronically ischemic skeletal muscle.

### 2.5. The Protein Expression of Hypoxia-Inducible Factor-1α (HIF-1α) and Vascular Endothelial Growth Factor (VEGF)

**Figure 5:**
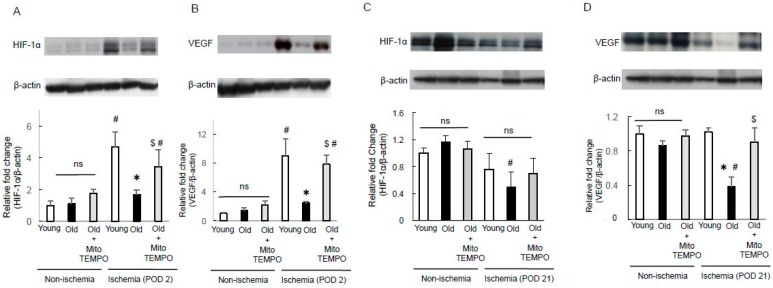
The protein expression of hypoxia-inducible factor-1α (HIF-1α) and vascular endothelial growth factor (VEGF). The expression of HIF-1α and VEGF were analyzed by Western blotting in the non-ischemic and ischemic (POD 2 and 21) skeletal muscles of young, old, and MitoTEMPO-treated old mice. Representative images of HIF-1α ((**A**,**C**) POD 2, 21, respectively) and VEGF ((**B**,**D**) POD 2, 21, respectively) and summarized findings after quantification of HIF-1α/β-actin and VEGF/β-actin. For POD 2, both HIF-1α and VEGF expression during ischemia were higher in young mice than in non-ischemic mice, but not in old mice. MitoTEMPO treatment upregulated HIF-1α and VEGF expression in the skeletal muscle of old mice. For POD 21, neither HIF-1α nor VEGF expression under ischemia were elevated compared to non-ischemic mice. HIF-1α expression did not differ among the three groups. The VEGF expression of aged skeletal muscle was lower than young muscle, and MitoTEMPO treatment preserved the expression of VEGF. The values are expressed as the means ± S.E.M. *n* = 10, each. * *p* < 0.05 vs. young mice, $ *p* < 0.05 vs. old mice, # *p* < 0.05 vs. non-ischemic skeletal muscle. ns indicates no significant difference.

HIF-1α and VEGF levels are known to decrease with aging [4,26]. According to previous reports, excessive oxidative stress can impair VEGF-induced angiogenesis in endothelial cells [27]. To examine the proangiogenic effect of mitochondrial superoxide scavenging, we examined the expression of HIF-1α and VEGF by Western blot analysis. In non-ischemic skeletal muscles, the expression of HIF-1α (Figure 5A) and VEGF (Figure 5B) did not differ among young, old, and MitoTEMPO-treated old mice. However, at POD 2 after ischemia, HIF-1α and VEGF expression (Figure 5A,B) were significantly induced in young mice. The elevation of HIF-1α and VEGF expression were suppressed in old mice, which were recovered by MitoTEMPO treatment. These data suggest that mitochondrial superoxide scavenging is associated with elevated HIF-1α and VEGF expression in old skeletal muscle. The expression of HIF-1α and VEGF in old mice at day 21 after ischemia were lower than those of non-ischemic old mice (Figure 5C,D). In ischemic conditions, the expression of HIF-1α in old mice was not different from that in young mice. In addition, the magnitude of HIF-1α was smaller than that in POD 2 in the same conditions. Therefore, we propose that the contribution of HIF-1α to angiogenesis is rare in the late phase of ischemia.

### 2.6. The Expression of p53 and Apoptotic Factors

**Figure 6:**
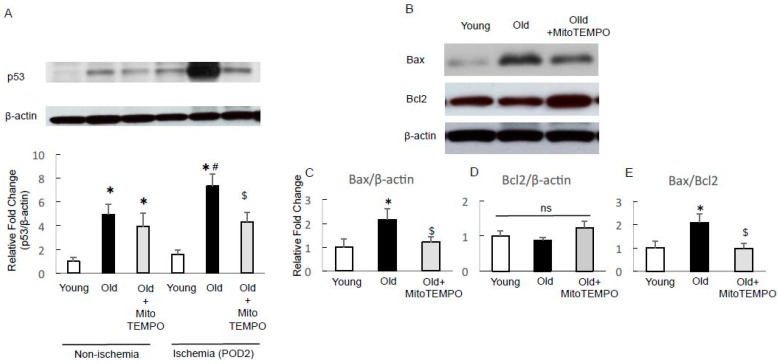
The protein expressions of p53, B-cell leukemia/lymphoma 2 (Bcl2) and Bcl2-associated X (Bax). The expression of p53 was analyzed by Western blotting in non-ischemic and ischemic (POD 2) skeletal muscle from young, old, and MitoTEMPO-treated old mice. The p53 expression was higher in old mice compared to young mice for non-ischemic and ischemic (POD 2) skeletal muscles. MitoTEMPO treatment attenuated the elevation of p53 after induction of ischemia (**A**). The expression of the proapoptotic factor Bax and the antiapoptotic factor Bcl2 was analyzed by Western blotting in the ischemic (POD 2) skeletal muscles of young, old, and MitoTEMPO-treated old mice. Representative images of Bax and Bcl2 (**B**) and the summarized findings after quantification of Bax/β-actin (**C**); Bcl2/β-actin (**D**) and Bax/Bcl2 (**E**). The Bax/Bcl2 ratio was significantly higher in old mice compared to young mice. MitoTEMPO treatment decreased the Bax/Bcl2 ratio to a similar level to that of young mice. The values are expressed as the means ± S.E.M. *n* = 10, each. * *p* < 0.05 vs. young mice, $ *p* < 0.05 vs. old mice, # *p* < 0.05 vs. non-ischemic skeletal muscle. ns indicates no significant difference.

The tumor suppressor p53 responds to hypoxia and is stabilized during severe hypoxia [28]. In severe hypoxia, p53 competes with HIF-1α to binding to p300, leading to HIF-1α downregulation [29]. Moreover, according to previous reports, oxidative mitochondrial damage causes p53 activation [30]. Taking into account our finding that decreasing mitochondrial ROS led to HIF-1α upregulation, we assumed that MitoTEMPO treatment could downregulate p53 expression. Thus, we examined whether MitoTEMPO treatment can attenuate the p53 expression in ischemic aged skeletal muscle. In non-ischemic skeletal muscle, the expression of p53 was higher in old mice than in young mice. MitoTEMPO treatment failed to reduce p53 in old mice. The p53 expression in ischemic muscle at POD 2 in old mice was markedly higher compared to young mice, which was attenuated by MitoTEMPO treatment (Figure 6A). A similar tendency was seen in the p53 expression of ischemic muscle on POD 21 (Figure S1). These findings suggest that mitochondrial ROS decrease in aged skeletal muscle attenuates p53 upregulation under ischemia. This mechanism may contribute to the upregulation of HIF-1α under ischemia. Apoptosis is a well-known anti-angiogenetic mechanism. According to previous reports, p53 mediates apoptosis by activating proapoptotic genes (*Bak*, *Bax*) and repressing antiapoptotic B-cell leukemia/lymphoma 2 (Bcl2) family genes [31,32]. Thus, we tested whether resolution of mitochondrial ROS could prevent ischemia-induced apoptosis. Western blot analysis revealed the elevated expression of Bcl2-associated X (Bax) and the Bax/Bcl2 ratio in old mice compared to young mice and the MitoTEMPO treatment decreased the expression of Bax and the Bax/Bcl2 ratio to the level of young mice (Figure 6B–E). Overall, these data suggest that attenuating mitochondrial ROS recovered the ischemia-induced apoptotic changes in aged skeletal muscles. The ratio of the weight of the ischemic hind limb and the gastrocnemius-soleus muscle (POD 21) per non-ischemic muscles were reduced in old mice compared to those in young and MitoTEMPO-treated old mice (Table S1), which may partially reflect the consequences of muscular apoptosis.

### 2.7. The Protein Expression of Peroxisome Proliferator-Activated Receptor γ Coactivator-1α (PGC-1α), Estrogen-Related Receptor α (ERRα), and Nuclear Respiratory Factor (NRF)-1

**Figure 7:**
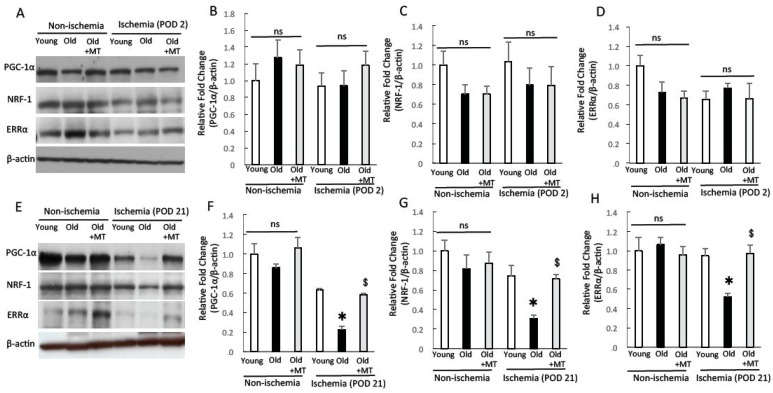
The protein expressions of peroxisome proliferator-activated receptor γ coactivator-1α (PGC-1α), nuclear respiratory factor (NRF)-1 and estrogen-related receptor α (ERRα). The expression of PGC-1α, NRF-1 and ERRα was analyzed by Western blotting in the non-ischemic and ischemic (POD 2 and 21) skeletal muscles of young, old, and MitoTEMPO-treated old mice. Representative images of PGC-1α, NRF-1 and ERRα for POD 2 (**A**) and POD 21 (**E**) and the summarized findings after quantification of PGC-1α/β-actin ((**B**,**F**) POD 2, 21, respectively), NRF-1/β-actin ((**C**,**G**) POD 2, 21, respectively) and ERRα/β-actin ((**D**,**H**) POD 2, 21, respectively). For POD 2, the expression of PGC-1α, NRF-1 and ERRα did not differ among the three groups. For POD 21, the expression of PGC-1α, NRF-1 and ERRα was lower in old mice than young mice. MitoTEMPO treatment effectively preserved PGC-1α, NRF-1 and ERRα expression to similar levels as young mice. MT indicates MitoTEMPO. The values are expressed as the means ± S.E.M. *n* = 10, each. * *p* < 0.05 vs. young mice, $ *p* < 0.05 vs. old mice. ns indicates no significant difference.

The transcriptional coactivators of PGC-1α have been identified as crucial regulators of mitochondrial biogenesis and function [33,34]. In addition, PGC-1α has been shown to mediate a HIF-1-independent pathway of angiogenesis in response to hypoxia [35]. Thus, we examined the effect of mitochondrial ROS scavenging for PGC-1α and its transcriptional factors, estrogen-related receptor α (ERRα) and nuclear respiratory factor (NRF)-1 expression under ischemia. In non-ischemic skeletal muscle, the expression of PGC-1α, ERRα and NRF-1 did not differ among three groups of mice (Figure 7A–D). We also could not find a significant difference between these factors in ischemic skeletal muscle at POD 2 (Figure 7A–D). Meanwhile, in ischemic skeletal muscles at POD 21, the expression of PGC-1α, NRF-1 and ERRα were lower in old mice than in young mice. MitoTEMPO treatment recovered the expression of PGC-1α, NRF-1 and ERRα to similar levels as young mice (Figure 7E–H). We found that mitochondrial superoxide scavenging preserved the expressions of PGC-1α and its transcriptional factors in aged skeletal muscles during prolonged ischemia, indicating the involvement of angiogenesis in the chronic phase of ischemia. This finding also supports reduced mitochondrial respiration in ischemic aged skeletal muscle at POD 21.

## 3. Discussion

Senescence is related to most forms of cardiovascular disease [1,2,3]. Our results demonstrate that ROS plays a crucial role in the age-associated impairment of blood flow, shedding light on the role of mitochondrial ROS in aging skeletal muscle. Moreover, we showed that mitochondrial ROS-targeted therapy for aging skeletal muscle attenuates mitochondrial DNA damage, mitochondrial dysfunction and apoptosis under ischemia, thus contributing to angiogenesis.

Our main findings are that administration of a mitochondria-targeted antioxidant, MitoTEMPO, leads the downregulation of p53 and the preservation of PGC-1α, which may be associated with angiogenesis genesis and mitochondrial function. The tumor suppressor p53 is upregulated in response to cellular stress, including DNA damage, oxidative stress and hypoxia [36]. Activated p53 interferes with hypoxia-sensing systems and degrades HIF-1α, leading to impaired VEGF expression [37]. In addition, p53 is the pivotal factor that induces apoptosis by recognizing external stimuli [31,32,38]. For example, in an ischemia/reperfusion model of aged rats, inhibition of p53 by pifithrin-α has a protective effect, thereby attenuating p53/Bax-mediated myocyte apoptosis during the early stages of ischemia [39]. In our study, p53 was upregulated by aging and ischemic stimuli, which seemed to lead to apoptosis and inhibit collateral growth through the HIF-1/VEGF axis. In contrast, MitoTEMPO treatment could effectively downregulate p53 expression. Taking into account the fact that ROS itself activates p53, decreasing ROS and DNA damage by mitochondrial-targeted antioxidants is likely the main reason for the downregulation of p53. Although p53 is thought to act as an anti-angiogenetic factor in contrast to VEGF, ischemic stimuli elevated p53 expression not only in old mice but also in young and old mice treated with MitoTEMPO. According to previous reports, p53 represses VEGF expression under continued hypoxic conditions. However, it can also positively regulate VEGF expression during the initial phase of hypoxia by binding to conserve sites in the VEGF promoter [40]. Thus, the elevation of p53 is crucial in the early phases of ischemia induction, and our results seem to be compatible with this previous report. PGC-1α also dramatically induces angiogenesis through a HIF-1-independent pathway [35]. PGC-1α binds to its transcriptional genes such as *ERRα* and *NRF*s, allowing for the induction of angiogenic genes and mitochondrial genes [41]. In our study, MitoTEMPO treatment could not elevate the PGC-1α expression in ischemic skeletal muscle (POD 2). Therefore, it is likely that mitochondrial ROS scavenging does not affect angiogenesis in the early phase of ischemia. However, in the late phase (POD 21), the expression of PGC-1α and its transcriptional factor were preserved by MitoTEMPO treatment. Several reports demonstrated that PGC-1α is induced by increased exercise training and physical activity, leading to angiogenesis and mitochondrial biogenesis [42,43]. In addition, recent studies have demonstrated that mitochondrial respiration or electron transport chain (ETC) activity show strong dependency on the physical activity level and a decline of skeletal muscle in aging causes impairment of exercise capacity [44,45]. According to the result that muscle volume in ischemic zone was lower in old mice compared to that in young mice, we speculate that aging exacerbates ischemia-induced muscle decline, which leads to impaired exercise capacity. In our data, lower expression of PGC-1α appeared in the ischemic muscle of old mice compared to that of young mice. Therefore, a low exercise capacity in the hind limb might partially contribute to the reduction of PGC-1α expression as well as the decrease in mitochondrial respiration and RCR value in skeletal muscle with prolonged ischemia of old mice. Likewise, mitochondrial antioxidant therapy would be a potential avenue to improve exercise capacity and sarcopenia in ischemic peripheral artery disease in aged patients; this requires further clinical studies. The relationship of mitochondrial respiration profile and collateral growth under ischemia remains to be investigated, although a previous report demonstrates that an improvement in the mitochondrial profile leads to restoration of coronary collateral growth [46]. However, in our results, the respiratory control ratio, indicating the efficiency of ATP production in mitochondria, was reduced in the ischemic muscle of old mice compared to young mice. Therefore, taking into account the fact that the ATP originating in mitochondria is critical for maintaining the energy status of the cell, an altered mitochondrial respiration profile may possibly attribute to collateral development in ischemic muscle.

There are a few limitations to this study. First, the current work did not show eNOS activity. eNOS is crucial for VEGF-triggered angiogenesis because NO stimulates VEGF, and eNOS also acts as an essential effector of downstream VEGF signaling for angiogenesis [47,48]. However, our previous study revealed that MitoTEMPO treatment recovered eNOS function and activity in the ischemic muscle of old mice [49]. Thus, preservation of eNOS activity by MitoTEMPO may contribute to improvements in age-related collateral development. Second, we did not demonstrate whether the effect of MitoTEMPO is dose-dependent. It has been reported that a low level of ROS is essential for angiogenesis through HIF-VEGF pathway signaling [14]. In our study, MitoTEMPO treatment did not suppress angiogenesis because MitoTEMPO treatment did not decrease mitochondrial H_2_O_2_ and superoxide in ischemic skeletal muscle to the level of non-ischemic mice. However, in the case of a higher dose of MitoTEMPO, angiogenesis under ischemia may be attenuated due to an insufficient amount of ROS. Third, we did not show mitochondrial respiration via complex І. However, it has been reported that the respiration of complex II is closely related in cell survival under hypoxia [50]. Therefore, we examined mitochondrial respiration via complex II. Moreover, we did not measure mitochondrial respiration in different muscle that is composed of fast-twitch muscle fibers (such as the extensor digitorum longus). Age-related changes in mitochondrial function vary in different muscle types [25], thus we cannot predicate whether our results apply to other muscle types. Further studies are needed to clarify the precise mechanisms of mitochondrial respiration in skeletal muscle following ischemia.

## 4. Materials and Methods

### 4.1. Animals

The investigations conformed to the Guide for the Care and Use of Laboratory Animals published by the U.S. National Institutes of Health (NIH Publication, 8th edition, 2011). Our research protocol was approved by the Fukushima Medical University Animal Research Committee (permit number 27058 (1 April 2015)), and all animal experiments were conducted in accordance with the guidelines of the Fukushima Medical University Animal Research Committee. All efforts were made to minimize animal Suffering. and old wild type C57BL/six male mice (Young; eight weeks (W), body weight (B.W.) 19.6 ± 0.2 g, Old; 80 W, B.W. 28.4 ± 0.3 g) were housed and bred in a room at 22 ± 3 °C, with a relative humidity of 50 ± 10% and a 12-h light–dark cycle*.* The mice were given food and water ad libitum.

### 4.2. Animal Experiments

The mice received a separate mini-pump for co-infusion of MitoTEMPO (Santa Cruz Biotechnology, Dallas, TX, USA) or saline. The mice were anesthetized by pentobarbital (50 mg/kg body weight, i.p.) and an osmotic mini-pump (ALZET micro-osmotic pump, model 1002, DURECT Co., Cupertino, CA, USA) was subcutaneously implanted. MitoTEMPO (180 µg/kg/day) and saline for the old mice and saline for the young mice were continuously infused. From the previous study concerning to the hemodynamic effect of MitoTEMPO, we determined 180 µg/kg/day of MitoTEMPO is a suitable dose [15,49]. Unilateral hind limb ischemia was induced by ligation of the left femoral artery from its origin just below the inguinal ligament under tribromoethanol anesthesia (200 mg/kg body weight, i.p.) at day 7 after pump implantation [51]. The sham operation was without femoral artery ligation but with skin incision in the right hind limb of ischemia-induced mice. The mice were sacrificed with isoflurane aspiration and a lethal dose of pentobarbital (60 mg/kg body weight i.p.) on POD 2 or 21, and examinations were performed. We selected POD 2 as early phase of ischemia, because the expressions of HIF-1α and VEGF were more remarkable than other days in our preliminary test.

### 4.3. Measurement of Hind Limb Perfusion

Scanning laser Doppler perfusion imaging (Moor Instruments, Wilmington, DE, USA) was used to record the hind limb perfusion under 1.125% isoflurane/O_2_ anesthesia. The ratio of ischemic to normal laser Doppler blood flow was measured before ligation and on POD 0, 1, 7, 14, and 21. The ratio of ischemic-to normal laser Doppler blood flow was gained by comparison of ischemic limb and contra-lateral limb in each measurement. Average perfusion was recorded from the plantar to inguinal surface, which indexes overall limb blood flow, and is expressed as the ischemic/non-ischemic ratio for assessing the variables, including ambient light and temperature [51].

### 4.4. Mitochondrial Isolation

Skeletal muscle mitochondria were isolated from the soleus of both ischemic and non-ischemic hind limbs based on previously reported protocols [52,53]. Each soleus was washed in mitochondria isolation buffer (MSHE: 70 mM sucrose, 210 mM mannitol, 5 mM 4-(2-hydroxyethyl)-1-piperazineethanesulfonic acid (HEPES), 1 mM ethylene glycol-bis(2-aminoethylether)-*N*,*N*,*N*′,*N*′-tetraacetic acid (EGTA), 0.5% fatty acid-free bovine serum albumin (BSA) (pH 7.2)), minced on ice and trypsinized for 2 min. Trypsinized muscles were homogenized using a Dounce homogenizer (Wheaton, Millville, NJ, USA) in cold MSHE buffer. Homogenates were centrifuged at 800× *g* for 10 min. Supernatants were removed and centrifuged at 8000× *g* for 10 min. Pellets were washed three times in MSHE buffer without BSA and the protein was estimated using the Bradford protein assay (Bio-Rad, Hercules, CA, USA). The mitochondrial pellet was resuspended at 10 μg/50 μL in mitochondria assay buffer (MAS: 70 mM sucrose, 220 mM Mannitol, 10 mM KH_2_PO_4_, 5 mM MgCl_2_, 5 mM HEPES, 1 mM EGTA, 0.2% fatty acid-free BSA (pH 7.2)) for measurement of mitochondrial respiration and H_2_O_2_ concentration.

### 4.5. Mitochondrial H_2_O_2_ Concentration Measurement

The mitochondrial H_2_O_2_ concentration was measured using an Apollo 4000 Free Radical Analyzer equipped with a 100 μm H_2_O_2_ electrode (WPI Co, Sarasota, FL, USA) as previously described [54,55]. The measurements were performed on hot plate at 34 °C with a reaction volume of 500 μL. Each sample contained MAS buffer (70 mM sucrose, 220 mM Mannitol, 10 mM KH_2_PO_4_, 5 mM MgCl_2_, 5 mM HEPES, 1 mM EGTA, 0.2% fatty acid-free BSA (pH 7.2)) and 50 μg of mitochondrial protein. Values were obtained at the point of a stable output signal and were converted to H_2_O_2_ concentration (μM/1 g of mitochondrial protein) using a predetermined H_2_O_2_ standard curve. To minimize errors, mitochondria were isolated on ice and the incubation time was stabilized.

### 4.6. Ultra-Performance Liquid Chromatography (UPLC)

Dihydroethidium (DHE) can be oxidized by oxidants, leading to the formation of other fluorescent products such as 2-hydroxyethidium (EOH) and ethidium. EOH is mainly generated by a reaction with superoxide. We performed UPLC using DHE to estimate superoxide levels in the gastrocnemius and soleus muscles as previously described [15] with modifications [56]. Ischemic and non-ischemic soleus 48 h after femoral artery ligation were immediately cut into 20-mg samples from the distal side and incubated with Krebs HEPES buffer containing 50 μM DHE at 37 °C for 30 min. The tissues were then washed with DHE in Krebs HEPES buffer, placed in 300 μL of cold methanol and homogenized. After centrifugation (×8000 rpm, for 10 min), 200 μL of supernatant was exsiccated by nitrogen gas, dissolved in 100 μL of 0.2% formic acid, and filtered (0.22 μm). The filtrate was then analyzed by UPLC. Separation of 2-hydroxyethidium, ethidium and DHE was performed using a Waters AQUITY UPLC H-class system with an AQUITY BEH C18 column (particle size 1.7 µm, φ 2.1 × 50 mm, Waters, Milford, MA, USA) at 40 °C.

### 4.7. Semi-Quantitative PCR for mtDNA Damage

mtDNA was isolated from 250 mg of hind limb skeletal muscle using a mtDNA extractor CT kit (Wako, Osaka, Japan). mtDNA damage was assayed by different length quantitative PCR [57,58]. The principle of this assay is that lesions in mtDNA introduced by oxidative damage can block polymerase progression during PCR, reducing product abundance when compared to the undamaged DNA. We used a long 10-kb mtDNA target for analyzing oxidative damage, compared to a short 127-bp target as control for mtDNA copy number. Different length qPCR amplifications were conducted in a 50 μL volume containing 15 μg of mtDNA in 15 μL and 35 μL PCR mastermix (5 μg BSA, 200 μM dNTPs, 20 pmol forward and reverse primers, 0.1 mM Mg, 1 U/μL Ex Taq, 3.5 μL 10× EX Taq buffer (Takara-bio, Kusatsu, Japan) and 31.5 μL nuclease-free water). The mouse large 10 kb mtDNA fragment primers were:

5′GCC AGC CTG ACC CAT AGC CAT AAT

5′GAG AGA TTT TAT GGG TGT AAT GCG.

Mouse small 127 bp mtDNA fragment primers were:

5′GCC AGC CTG ACC CAT AGC CAT AAT

5′GCC GGC TGC GTA TTC TAC GTT A.

The reactions were initiated with the hot start method. For long fragment PCR amplification, DNA was denatured initially at 75 °C for 2 min and 95 °C for 1 min, and then the reaction underwent 16 cycles of 94 °C for 15 s, 64 °C for 12 min, with a final extension of 72 °C for 10 min. For small fragment PCR amplification, DNA was denatured initially at 75 °C for 2 min and 95 °C for 15 s, followed by 23 cycles of 94 °C for 30 s, 64 °C for 45 s, 72 °C for 45 s, and then 72 °C for 10 min. PCR products were quantified by the PicoGreen ds DNA assay kit (Thermo Fisher Scientific, Waltham, MA, USA) as per the manufacturer’s protocol. The amplification of the long product (A_L_) was normalized to the short product (A_S_) for each sample, resulting in a relative amplification ratio. Using the “zero class” of a Poisson equation, the lesion frequency per fragment at a particular dose was determined: λ = ln A_L_/A_S_ (λ = the average lesion frequency) [59].

### 4.8. Measurement of Mitochondrial Respiration

Mitochondrial oxygen consumption was measured using the XF24 instrument as reported previously [53]. A detailed description of measurement of mitochondrial respiration protocols is in the supplementary materials online. We plated 10 μg of skeletal muscle mitochondria in each well of the XF24 v7 plate in a volume of 50 μL containing MAS buffer with 10 mM succinate and 2 μM rotenone. After centrifugation (for 20 min at 2000 rpm), 450 μL of MAS containing succinate and rotenone was added and incubated at 37 °C for 10 min. The plate with mitochondria was introduced into the XF machine and assayed using the protocol developed by Rodgers et al [53]. In this protocol, 50 μL of ADP (40 mM), 55 μL of oligomycin (2 μg/mL), 60 μL of carbonyl cyanide-p-trifluoromethoxy-phenylhydrazone (FCCP, 40 μM), and 65 μL of antimycin A (40 μM) were injected into each sample in sequence. Therefore, the final concentrations were: ADP 4 mM, oligomycin 2.5 μM, FCCP 4 mM and antimycin A 4 μM. Oxygen consumption rates (pmols oxygen per minute) were monitored in real time and we could determine basal respiration (state 2 respiration), phosphorylating respiration in the presence of ADP (state 3 respiration), resting respiration (state 4 respiration), maximal uncoupling respiration in the presence of FCCP (state 3u respiration) and electron transport chain-unrelated respiration in the presence of complex Ш inhibitor antimycin A. The respiratory control ratio (RCR) was determined by dividing the rate of state 3 respiration by state 4 respiration.

### 4.9. Immunohistochemistry

Bilateral gastrocnemius and soleus muscles were dissected and snap-frozen in a bath of liquid nitrogen on post-operative day 21. Muscles were sectioned on a cryostat to a thickness of 7 μm and fixed in 100% methanol and H_2_O_2_. Immunohistostaining was performed with rabbit anti-mouse CD31 antibody (Santa Cruz Biotechnology) and an anti-rabbit Ig horseradish peroxidase detection kit (Nichirei biosciences, Chuo, Tokyo, Japan). The capillary count was performed under light microscopy (magnification ×200). The number of capillaries per muscle fiber was measured in 5 randomly chosen fields from three different sections in each tissue block [60]. For demonstrate representative morphology of ischemic muscles, sections were stained with Hematoxylin and Eosin (H & E).

### 4.10. Western Blotting

Frozen gastrocnemius and soleus muscles were pulverized in liquid nitrogen and suspended in lysis buffer with protease inhibitor. After quantifying the protein concentrations using the Bradford protein assay (Bio-Rad), equal amounts of protein (20 μg/sample) were analyzed by 10% or 15% sodium dodecyl sulfate polyacrylamide gel electrophoresis (SDS-PAGE) gels depending on the molecular weight. The gels were transferred onto nitrocellulose membranes. Membranes were blocked in Tris-buffered saline Tween 20 (TBST) containing 5% BSA and immunoblotted using the following primary antibodies: anti-p53 (PAb122) antibody (Enzo Life Science, Inc., Farmingdale, NY, USA, 1:1500), anti-PGC1 α (ab54481) antibody (Abcam, Cambridge, UK, 1:1500), anti-HIF-1α (#14179) antibody (Cell Signaling, Danvers, MA, USA, 1:1000), anti-Bax (2772) antibody (Cell Signaling, 1:1000), anti-Bcl-2 (2876) antibody (Cell Signaling, 1:1000), anti-VEGF (sc-507) antibody (Santa Cruz Biotechnology, 1:1000), anti-β-Actin (sc-47778) antibody (Santa Cruz Biotechnology, 1:10,000), anti-ERR α (EPR46Y)) antibody (Novus Biologicals, Littleton, CO, USA, 1:1000), and anti-NRF1 (200-401-869) antibody (Rockland, Limerick, PA, USA, 1:1000). Membranes were then washed in TBST and incubated with the appropriate anti-mouse or anti-rabbit horseradish peroxidase-linked secondary antibody (Santa Cruz Biotechnology, 1:10,000). Antibody-bound protein was visualized using the enhanced chemiluminescent method. The relative intensities of the protein bands were quantified using NIH Image J, version (1.48) (Scion Image, NIH, Bethesda, MD, USA). Data were normalized to β-actin and are expressed as a fold change to the non-ischemic skeletal muscle of young mice in the same period for each analysis.

### 4.11. Statistical Analysis

Values are expressed as the means ± S.E.M. One-way repeated measures ANOVA with Tukey’s post hoc test was used for hind limb blood flow data. For all other data sets, one-way factorial ANOVA with Tukey’s post hoc test were carried out to compare multiple groups. A *p* value less than 0.05 were considered to be statistically significant. All statistical analyses were performed using SPSS software program (ver.23.0, IBM, Armonk, NY, USA).

## 5. Conclusions

**Figure 8:**
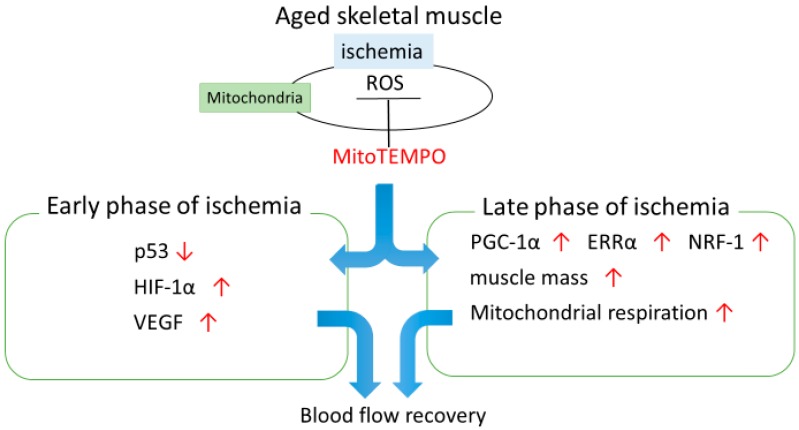
The role of mitochondrial ROS in the regulation of blood flow recovery with ischemia in aging. In aged muscle, mitochondrial ROS induced by ischemia impairs blood flow recovery. Mitochondrial superoxide scavenging improves blood flow related to downregulation of p53, upregulation of HIF-1α and VEGF in the early phase of ischemia, and preservation of PGC-1α, NRF-1, and ERRα in the late phase of ischemia. Scavenging of mitochondrial superoxide with MitoTEMPO is an effective means to preserve blood flow following limb ischemia. Red arrows pointing upwards indicate increase, pointing down indicate decrease of the protein expression, muscle mass and mitochondrial respiration in MitoTEMPO-treated aged muscle compared to non-treated one.

Our findings suggest that mitochondrial ROS scavenging contributes to the attenuation of age-dependent mitDNA damage and excessive ROS elevation after ischemia. We also demonstrated that mitochondrial ROS induced by ischemia impairs blood flow recovery, and mitochondrial superoxide scavenging improves collateral growth related to downregulation of p53, upregulation of HIF-1α and VEGF in the early phase of ischemia, and preservation of PGC-1α, NRF-1, and ERRα in the late phase of ischemia (Figure 8). Overall, treatment of mitochondria-targeted antioxidants can be an effective therapy to improve quality of life and outcomes in patients of critical limb ischemia.

## Supplementary Materials

Supplementary materials can be found at www.mdpi.com/1422-0067/18/9/1897/s1.

## Abbreviations

PGC-1α	Peroxisome proliferator-activated receptor γ coactivator-1α
NRF	Nuclear respiratory factor
ERRα	Estrogen-related receptor α
ROS	Reactive oxygen species
MitoTEMPO	2-(2,2,6,6-Tetramethylpiperidin-1-oxyl-4-ylamino)-2-oxoethyl) triphenylphosphonium chloride monohydrate
H2O2	Hydrogen peroxide
OCR	Oxygen consumption rate
RCR	Respiratory control ratio
FCCP	p-Trifluoromethoxyphenylhydrazone
UPLC	Ultra-performance liquid chromatography analysis
POD	Post-operative day
PAD	Peripheral artery disease
HEPES	4-(2-Hydroxyethyl)-1-piperazineethanesulfonic acid
EGTA	Ethylene glycol-bis(2-aminoethylether)-*N*,*N*,*N*′,*N*′-tetraacetic acid
MAS	Mitochondrial assay solution
ADP	Adenosine 5′-diphosphate sodium
TBST	Tris-buffered saline Tween 20
VEGF	Vascular endothelial growth factor
ETS	Electron transport system
HIF-1α	Hypoxia-inducible factor-1α
Bcl2	B-cell leukemia/lymphoma 2
Bax	Bcl2-associated X
BSA	Bovine serum albumin
SDS-PAGE	Sodium dodecyl sulfate polyacrylamide gel electrophoresis
