# Supplementary material for: Mitochondrial-Targeted Antioxidant Maintains Blood Flow, Mitochondrial Function, and Redox Balance in Old Mice Following Prolonged Limb Ischemia
Source: Int J Mol Sci. 2017 Sep 4;18(9):1897. doi: 10.3390/ijms18091897 (PMC5618546; doi:10.3390/ijms18091897)
Supplement: Supplementary file 1 [file ijms-18-01897-s001.pdf]

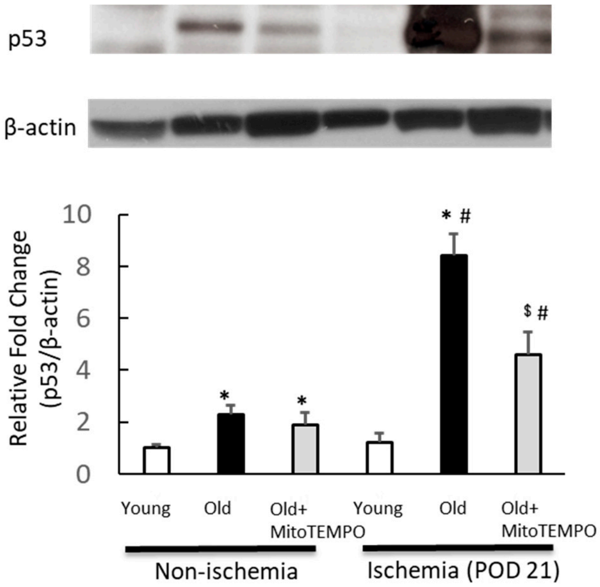

Figure S1. The expression of p53 was analyzed by western blotting in non-ischemic and ischemic (POD 21) skeletal muscle from young, old and MitoTEMPO-treated old mice. The p53 expression was higher in old mice compared to young mice for non-ischemic and ischemic (POD 21) skeletal muscles. MitoTEMPO treatment attenuated the elevation of p53 after induction of ischemia. The values are expressed as the means±S.E.M. n=6, each. *P<0.05 vs. young mice, $P<0.05 vs. old mice, #P<0.05 vs. non-ischemic skeletal muscle.

Table S1. Body weight of mice and weight of hind limb, gastrocnemius and soleus muscles. The ratio of ischemic hind limb (POD 21) to non-ischemic hind limb and the ratio of ischemic gastrocnemius and soleus muscles (POD 21) to non-ischemic gastrocnemius and soleus muscles were lower in old mice than young and MitoTEMPO-treated old mice. The values are expressed as the means±S.E.M. n=10, each. *P<0.05 vs. young mice, $P<0.05 vs. old mice.

		**Young mice**	**Old mice**	**MitoTEMPO-treated old mice**
B.W. (g)		19.6±0.2	28.2±0.2*	28.7±0.3*
hind limb (mg)	ischemia	955.0±21.4	887.8±33.5	1047.2±31.0
	Non-ischemia	1096.3±9.9	1230.5±23.7	1264.4±47.5
	Ischemia/non-ischemia	0.87±0.01	0.72±0.02*	0.83±0.02 $
Gastrocnemius-soleus muscle (mg)	ischemia	62.7±5.6	69.4±2.8	87.5±8.6
	Non-ischemia	75.4±4.0	118.3±2.0	115.1±5.4
	Ischemia/non-ischemia	0.82±0.03	0.58±0.02*	0.75±0.04 $
